# Gut microbial diversity, inflammation, and oxidative stress are associated with tacrolimus dosing requirements early after heart transplantation

**DOI:** 10.1371/journal.pone.0233646

**Published:** 2020-05-29

**Authors:** Douglas L. Jennings, Bruno Bohn, Amelia Zuver, Duygu Onat, Maureen Gaine, Eugene Royzman, Jonathan Hupf, Danielle Brunjes, Farhana Latif, Susan Restaino, Arthur R. Garan, Veli K. Topkara, Hiroo Takayama, Koji Takeda, Yoshifumi Naka, Maryjane Farr, Renu Nandakumar, Anne-Catrin Uhlemann, Paolo C. Colombo, Ryan T. Demmer, Melana Yuzefpolskaya

**Affiliations:** 1 Department of Pharmacy Practice, Long Island University, New York, New York, United States of America; 2 Department of Pharmacy, NewYork-Presbyterian Hospital Columbia University Medical Center, New York, New York, United States of America; 3 Division of Epidemiology and Community Health, School of Public Health, University of Minnesota, Minneapolis, MN, United States of America; 4 Division of Cardiology, Department of Medicine, NewYork-Presbyterian Hospital, Columbia University, New York, New York, United States of America; 5 Division of Cardiothoracic Surgery, Department of Surgery, NewYork-Presbyterian Hospital, Columbia University, New York, New York, United States of America; 6 Biomarkers Core Laboratory, Irving Institute for Clinical and Translational Research, NewYork Presbyterian Hospital, Columbia University, New York, New York, United States of America; 7 Department of Medicine, Division of Infectious Diseases and Microbiome and Pathogen Genomics Core, NewYork-Presbyterian Hospital, Columbia University, New York, New York, United States of America; 8 Division of Epidemiology, Mailman School of Public Health, Columbia University, New York, New York, United States of America; University of California Los Angeles, UNITED STATES

## Abstract

**Introduction:**

Effective tacrolimus (TAC) dosing is hampered by complex pharmacokinetics and significant patient variability. The gut microbiome, a key mediator of endotoxemia, inflammation and oxidative stress in advanced heart failure (HF) patients, is a possible contributor to interindividual variations in drug efficacy. The effect of alterations in the gut microbiome on TAC dosing requirements after heart transplant (HT) has not been explored.

**Methods:**

We enrolled 24 patients (mean age = 55.8 ±2.3 years) within 3 months post-HT. Biomarkers of endotoxemia ((lipopolysaccharide (LPS)), inflammation (tumor necrosis factor-α (TNF-α)) and oxidative stress (8,12-iso-Isoprostane F-2alpha-VI) were measured in 16 blood samples. 22 stool samples were analyzed using 16S rRNA sequencing. TAC dose and serum trough level were measured at the time of stool and blood collection. TAC doses were reported in mg/kg/day and as level-to-dose (L/D) ratio, and categorized as **≤** vs. > median.

**Results:**

The median TAC dose was 0.1 mg/kg/day and L/D ratio was 100.01. Above the median daily weight-based TAC dose was associated with higher gut microbial alpha diversity (p = 0.03); similarly, TNF-α and 8,12-iso-Isoprostane F-2alpha-VI levels were lower and LPS levels were higher in the above median TAC group, although these findings were only marginally statistically significant and dependent on BMI adjustment. We observed n = 37 taxa to be significantly enriched among patients with > median TAC dose (all FDR<0.05), several of which are potential short-chain fatty acid producers with anti-inflammatory properties, including taxa from the family *Subdoligranulum*.

**Conclusions:**

Our pilot study observed gut microbial alpha diversity to be increased while inflammation and oxidative stress were reduced among patients requiring higher TAC doses early after HT.

## Introduction

Tacrolimus (TAC) is the cornerstone of immunosuppression in heart transplant (HT) recipients [1]. Achieving and sustaining therapeutic TAC levels are essential in preventing cellular rejection in the early post-HT period [2,3]. Unfortunately, owing to its unpredictable pharmacokinetic profile and significant intra- and inter-patient variability, estimating accurate TAC dosing requirements and maintaining therapeutic levels is challenging. In the early post-transplant period, many factors can influence TAC dosing, including age, race, body habitus, anemia, liver dysfunction, malnutrition, changes in bowel functions such as ileus/diarrhea, drug-drug interactions, and genotypic aberrations (i.e. CYP3A5 carrier status) [4].

The interplay between gut microbial diversity and immunosuppression drug dosing has garnered recent interest in the solid-organ transplant community. An imbalance of microbial communities in the gut, commonly referred to as gut dysbiosis, may disrupt the intestinal lining and alter drug absorption.This theory is supported by data showing a link between diarrhea and elevated TAC levels, which is thought to be related to downregulation of intestinal cytochrome P4503A4 and P-glycoprotein activity [5]. Early animal models in rats [6] and mice [7] have explored the relationship between TAC dosing and the composition of the gut microbiota and suggest a corrleation. While these animal models are provocative, the first study in humans was by Lee et al., who evaluated 19 kidney recipients and found that gut microbial composition was associated with TAC dosing requirements early after transplant [8]. This association has never been evaluated in heart transplant (HT) recipients.

Our group has previosuly shown that dysbiotic gut microbial community is present among advanced heart failure (HF) patients, and persists after HT [9]. These changes are associated with activation of multiple inflammatory and/or oxidative stress pathways as well as elevated levels of circulating endotoxins such as lipopolysaccharides (LPS), which is produced by Gram-negative bacteria [9]. Alterations in the microbial composition may lead to intestinal epithelial barrier dysfunction and increased gut permiability, i.e. ‘the leaky gut”, allowing the translocation of pro-inflammatory bacteria and its bi-products into the peripheral circulation.

Presently, we investigate the variation in gut microbial communities, and levels of endotoxemia, inflammation and oxidative stress in a cohort of patients within the first 3 months after HT. We tested the following specific hypotheses cross-sectionally: 1) TAC dosing requirements are associated with microbial diversity; 2) TAC dosing requirements are related to levels of endotoxemia, inflammation and oxidative stress; and 3) specific microbial signatures are associated with TAC dosing requirements in the early post-transplant setting.

## Materials and methods

### Study population

This was a single center cross-sectional study of adult patients who underwent HT between April 2017 and August 2018 and had stool and/or blood sampling available within the first 3 months after HT. This time frame was chosen as patients in the first 3 months post HT will have similar TAC serum trough goal levels as outlined below. Patients were enrolled in the outpatient setting during routine clinical visits or the index hospitalization for HT surgery. Demographic information (age, sex, race/ethnicity), clinical characteristics, body mass index (BMI), and smoking status, as well as concomitant drug therapy were extracted from electronic medical records. The Columbia University Irving Medical Center (CUIMC) Institutional Review Board approved the study protocol. All participants signed written informed consent.

### Immunosuppression protocol

All patients included in this study received sequential immunosuppression in accordance with the center’s protocol consisting of induction with high dose methylprednisolone, which was followed by triple maintenance therapy with TAC, mycophenolate mofetil, and prednisone taper. Immediate-release TAC was dosed twice daily and started on post-operative day 1, dosages were initially weight-based and subsequently adjusted to maintain initial target levels of 10 to 12 ng/mL for the first 3 months. Mycophenolate mofetil was started at 1500 mg twice daily on the day of surgery. All patients received antifungal (nystatin suspension), *Pneumocystis jiroveci* (trimethoprim/sulfamethoxazole or dapsone), and antiviral (valganciclovir) post-transplantation prophylaxis for at least 6 months.

### Measurement of TAC levels

TAC levels and doses were gathered at the time of stool and blood sample collection. For evaluating the outcomes of interest, patients were divided into two groups according to their mg/kg/day TAC requirements: *low-dose* (below the cohort median) and *high-dose* (above the cohort median). Each outcome was also analyzed using dose-normalized trough levels (trough Level/Dose or L/D ratio [ng/mL]/[mg/kg/24h]), whereby *low-* and *high-*dose groups were again defined using median dichotomization. TAC levels were measured at New-York Presbyterian Hospital–CUIMC clinical laboratory using Chemiluminescent Microparticle Immunoassay (CMIA). The lower limit for detection of TAC was 2 ng/mL.

### Measurements of plasma and serum biomarkers

Biomarkers of endotoxemia (lipopolysaccharide [LPS]), inflammation (tumor necrosis factor-α [TNF-α)], and oxidative stress (8,12-iso-Isoprostane F-2alpha-VI levels) were measured in 16 samples collected and processed during a clinic visit or the inpatient setting. Serum TNF-α was assessed using a high sensitivity Enzyme Linked Immunoassay (ELISA) kit (RD systems, Minneapolis, MN). Plasma LPS was measured using a LAL chromogenic endotoxin quantitation kit (Pierce Thermoscientific, Rockford, IL). Plasma 8,12-iso-Isoprostane F-2alpha-VI levels was measured in butylated hydroxytoluene treated human plasma samples using LC-MS/MS on Acquity UPLC-Xevo TQS (Waters, Milford, MA).

### Stool collection

Patients who were enrolled in the inpatient setting provided stool samples in sterile stool hats using a protocol similar to the Human Microbiome Project [10] After collection, stool samples were stored at 4°C andtransferred to the CUIMC Microbiome Core Lab within 12 hours. Ambulatory patients were provided with a stool collection kit that included ice-packs and FedEx return packaging during routine clinic visits. Stool samples were shipped to the Core lab after collection by patients at home. In the Core lab, stool was processed, aliquoted and stored at -80°C. A total of 22 stool samples were collected.

### DNA extraction

DNA was extracted from stool using the MagAttract PowerSoil DNA Kit on an Eppendorf epMotion 5075 Liquid Handling Workstation.16S rRNA sequencing was performed on all stool samples. For bacterial sequencing, we amplified the V3-V4 regions of the 16S rRNA gene using standard primers with Illumina Nextera adaptors (Illumina, Madison WI). PCR products were purified using Agencourt AMPure XP beads (Beckman Coulter, Jersey City, NJ) and quantified using the Quant-iT Broad Range dsDNA Assay kit (Thermo Fisher Scientific, Waltham, MA). Libraries were normalized and pooled with a 10% PhiX spike and sequenced on an Illumina MiSeq with a v3 kit. Negative controls were included on all sequencing runs.

### Statistical analysis

Demultiplexed sequence files were processed and operationalized in R version 3.5.2, using the DADA2 pipeline (version 1.10.1) to identify exact sequence variants (ESVs) [11,12]. Reads were truncated at forward and reverse lengths of 260 and 220. We utilized a published bioinformatics workflow that utilizes DADA2 to identify ‘exact sequence variants’ and assigned taxonomy using the Silva Projects version 128 release. Descriptive statistics for the numer of sequence reads per sample were: minimum = 411, maximum = 43,775, median = 12,713, mean = 15,361.95 ± 12455.97. 16S analyses were carried out–without filtering low prevalence ESVs–using the Phyloseq package version 1.30.0. Alpha diversity (i.e., number and distribution of bacterial taxa within samples) was defined using the Shannon Index and the number of observed ESVs (we present alpha diversity results based on both rarefied (400 sequence reads) and nonrarefied data). We used a generalized linear regression model to regress Shannon index on TAC group. The p-value was derived from the type III sum of squares F-value. Analysis of beta diversity (i.e., microbial community membership similarity according to TAC dosing requirements) utilized principal coordinates analysis (PCoA) of the Bray-Curtis dissimilarity index and PCoA plots of the first 2 principal coordinates were plotted according to TAC dosing requirements. PERMANOVA analysis was conducted to identify whether patient categories explained variation in microbial communities and statistical significance was determined using the Adonis test. The DESeq2 package [13] version 1.26.0 was used to evaluate whether specific taxa (ESVs) differed by TAC dosing requirements. A false discovery rate (FDR) was used to correct for multiple comparisons. DESeq2 analysis was also conducted adjusting for body mass index (BMI). BMI was abstracted from medical records and dichotomized as overweight and non-overweight, using a cut-off of 25 kg/m^2^. Relative abundance of the taxa found to be statistically significantly different by TAC dosing in both DESeq2 analyses were presented. Microbiota diversity measures, phylum level relative abundance (arcsine square root transformed) as well as biomarkers of endotoxemia, inflammation and oxidative stress were regressed on TAC dosing requirements in generalized linear regression models.

## Results

A total of 24 HT patients were included in this analysis: 22 had stool samples and TAC levels collected, while 16 had at least one blood biomarker available. Baseline demographics and background medication use were similar between the *low-* and *high*-dose groups, with the exception of a slightly higher BMI in the *low*-dose group (Table 1). The average times from HT to sample collections are summarized in S1 Table. The median TAC dose was 0.1 mg/kg/day, while the median dose-normalized ratio was 100.01. The median TAC dose among below and above median groups were 0.07 and 0.18 mg/kg/day, respectively. The median TAC levels among below and above median groups were 65.6 and 166.3 ng/mL, respectively.

**Table 1:** Baseline demographic and clinical characteristics.

Demographics	Total group	Low-group	High-group	P-value
	N = 24	≤ Median TAC dose	> Median TAC dose	
		N = 12	N = 12	
Age at HT (years)	55.8 ±2.3	55.2 ±3.6	56.3 ±3.2	0.82
Male (n, %)	21 (87.5)	10 (83.3)	11 (91.7)	1.0
Race				0.12
White (n, %)	15 (62.5)	10 (83.3)	5 (41.7)	
Black (n, %)	7 (29)	2 (16.7)	5 (41.7)	
Other (n, %)	2(8)	0	2 (16.7)	
BMI, Mean ± SE	25.9 ±0.9	27.7 ±1.5	24.1 ±0.7	0.05
Diabetes (n, %)	10 (41.7)	5 (41.7)	5 (41.7)	1.0
AFib (n, %)	13 (54.1)	8 (66.7)	5 (41.7)	0.41
Hyptertension (n, %)	17 (70.8)	8 (66.7)	9 (75)	1.0
LVAD prior to HT (n, %)	18 (75)	8 (66.7)	10 (83.3)	0.64
Time post-transplant (days)	63.5 ±5.9	65.1±8.2	61.8±8.8	0.79
**Laboratory Values**				
Creatinine, mg/dL	1.34 ±0.11	1.32 ±0.17	1.37 ±0.14	0.74
AST, U/L	26.9 ±2.7	29.3 ±3.8	23.6 ±3.5	0.37
Total Bilirubin, mg/dL	0.56 ±0.11	0.48 ±0.11	0.66 ±0.22	0.35
**Medications**				
Mycophenolate Mofetil (n, %)	20 (83.3)	8 (66.7)	12 (100)	0.09
Nystatin (n, %)	21 (87.5)	9 (75)	12 (100)	0.22
Valgancyclovir (n, %)	11 (45.8)	5 (41.7)	6 (50)	1.0
Trimethoprim/ sulfamethoxazole (n, %)	16 (66.7)	8 (66.7)	8 (66.7)	1.0
Dapsone (n, %)	7 (29.2)	2 (16.7)	5 (41.7)	0.37
Antibiotics (n, %)*	22 (91.6)	11 (91.6)	11 (91.6)	1.0
Aspirin (n, %)	20 (83.3)	10 (83.3)	10 (83.3)	1.0
Loop Diuretics (n, %)	12 (50)	6 (50)	6 (50)	1.0
Statins (n, %)	21 (87.5)	9 (75)	12 (100)	0.21
Proton Pump Inhibitors (n, %)	14 (58.3)	7 (58.3)	7 (58.3)	1.0

*Included only antibiotics for treatment

Values are presented as mean± standard error unless specified. T-Test or Chi-square/Fisher’s exact performed where appropriate. HT = heart transplant, LVAD = left ventricular assist device, BMI = body mass index, AST = aspartate aminotransferase, Afib = atrial fibrillation.

### Gut microbiota diversity and TAC dosing

**Fig 1:**
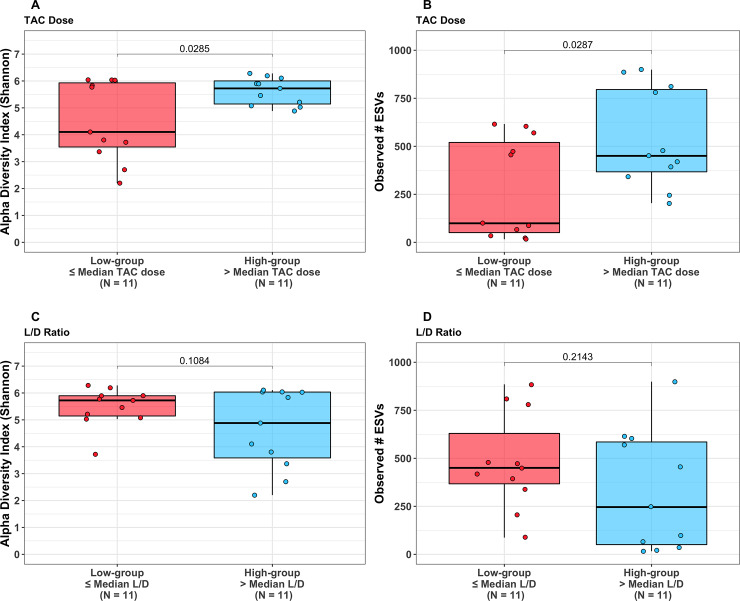
Measures of alpha-diversity (Shannon Index and number (#) of observed operationalized taxonomic units (ESVs)) by ≤ vs. > median weight adjusted TAC dose (1A and 1B) and by ≤ vs. > median Level/Dose (L/D) ratio (1C and 1D).

**Fig 2:**
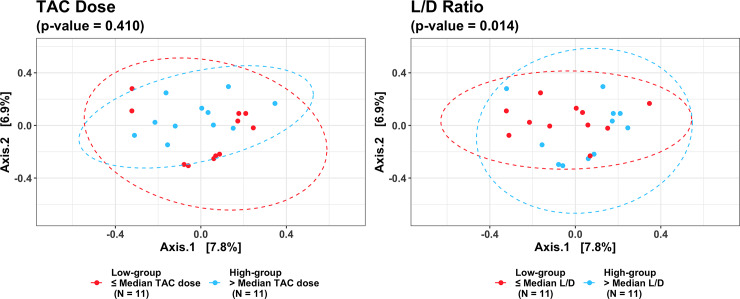
**Principle coordinates, derived from the Bray-Curtis dissimilarity index, plotted according to** ≤ **vs. > median weight adjusted TAC dose (left) and** ≤ **vs. > median Level/Dose (L/D) ratio (right).** Ellipses represent a 95% confidence interval for the data, assuming a normal distribution.

**Fig 3:**
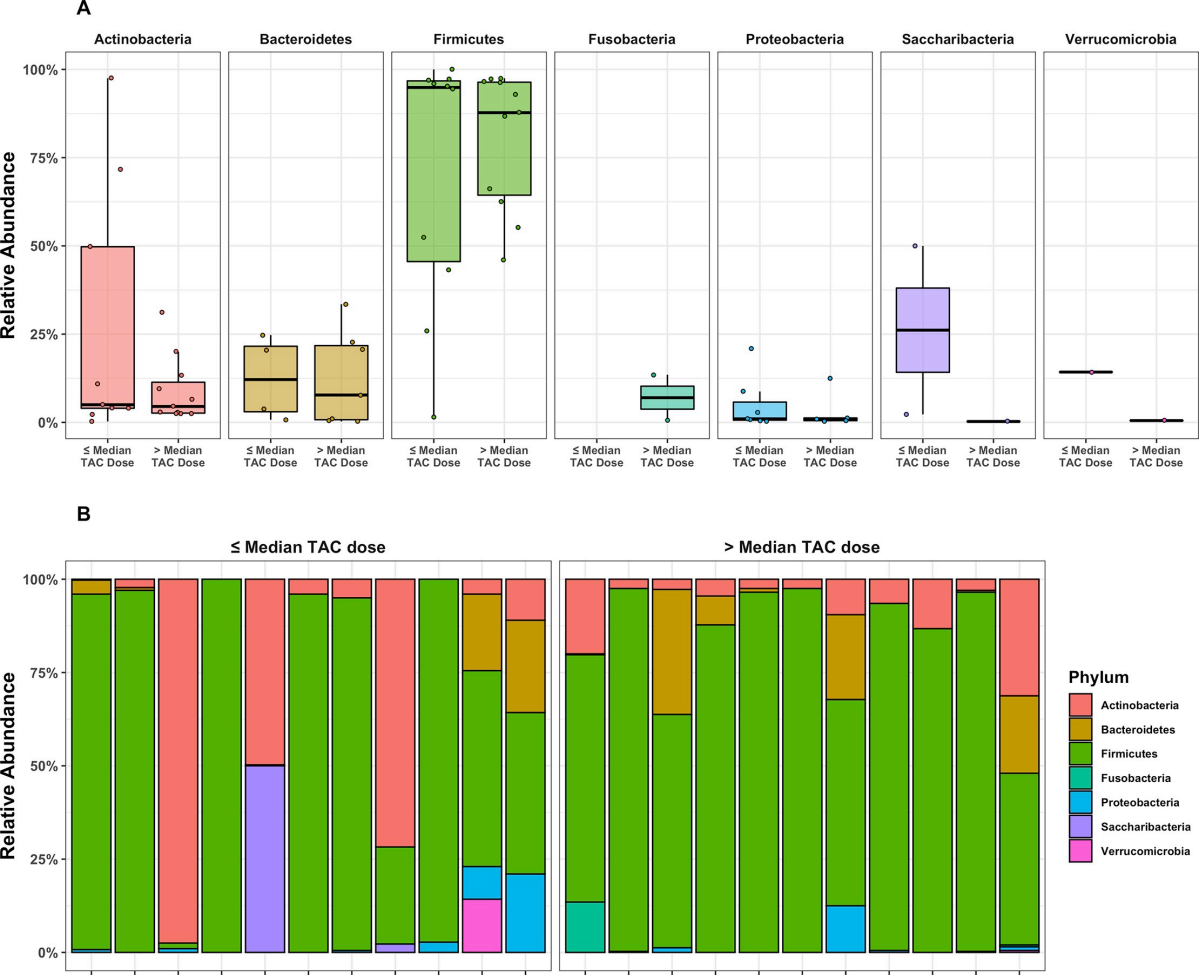
Top: Relative abundance of phyla per sample in ≤ vs > median weight adjusted TAC dose. Bottom: phyla composition by sample. No statistically significant difference was observed in phyla relative abundance by TAC dose group.

**Fig 4:**
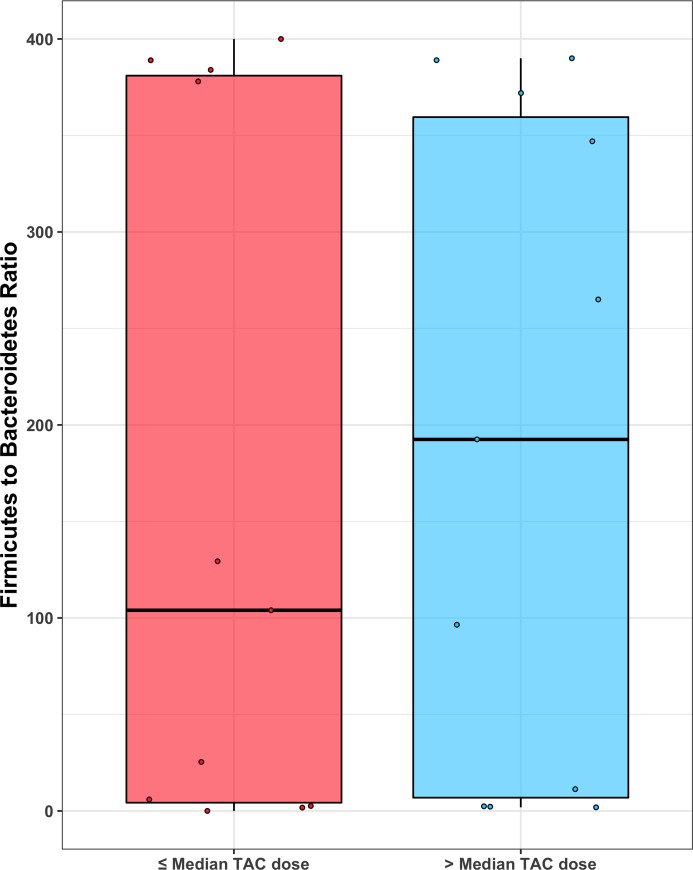
Firmicutes to Bacteroidetes ratio (using rarefied data) by TAC group. There is no statistical significance (p-value = 0.76) and the mean values are similar (165 and 188, respectively).

**Fig 5:**
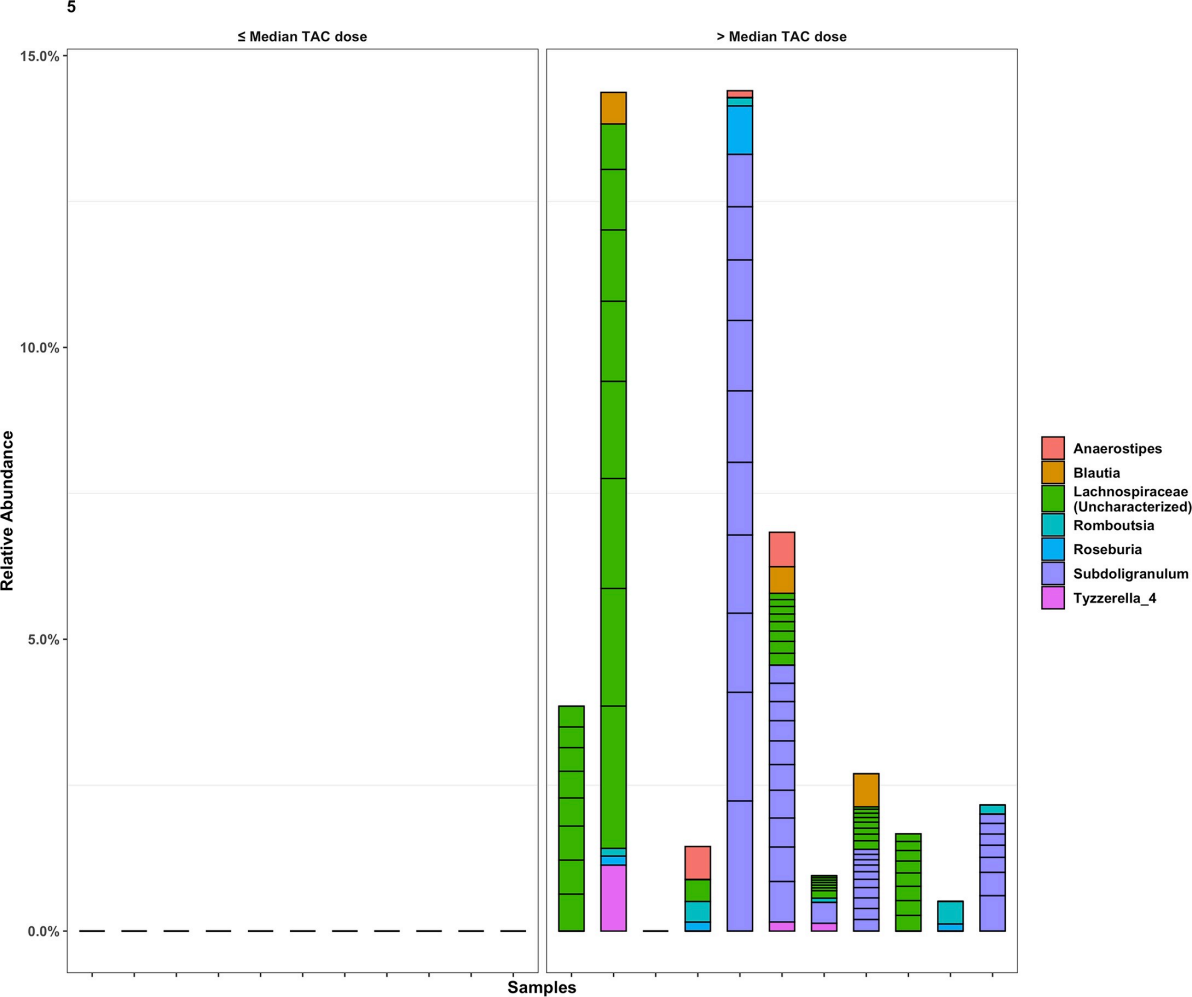
Stacked bar chart showing relative abundance of ESVs observed to be statistically significantly different between patients with ≤ vs. > median weight adjusted TAC dose. Taxa shown signififcantly differed in both adjusted and unadjusted analyses. Segments within a stack correspond to a single ESV and are colored according to genera. ESVs that did not significantly differ between TAC dose group are not displayed.

Mean values of the non-rarefied Shannon index and # of observed ESVs for the overall cohort were 5.06 and 406.54, respectively. Mean values of the Shannon index and # of observed ESVs after rarefication were 4.62 and 169.14. The following results are based on nonrarefied data as the rarefied and nonrarefied values were strongly correlated (p<0.0001) (S1 Fig). Patients in the *low*-dose tacrolimus group had a significantly lower alpha diversity metrics (Shannon index and # of observed ESVs) as compared to the *high*-dose group; these findings were consistent when using both weight-based dosing (mg/kg/day) or the L/D ratio (Fig 1). These findings were unchanged after adjusting for the difference in BMI using linear regression, and were consistent between the rarified and non-rarified data (S2 and S3 Figs) Fig 2 visualizes variation in microbial community composition (Beta diversity) between *low*- and *high*-dose cohorts by both mg/kg/day and L/D ratio. In phylum level analyses, Bacteroidetes and Firmicutes were the predominant microbes in both groups (Fig 3); while the ratio of Firmicutes to Bacteroidetes was numerically higher in the *high*-dose TAC group, this difference was not statistically significant (Fig 4). Results for other phyla are shown in Fig 3. We observed 37 taxa to be significantly enriched among patients in the *high*-dose group (all FDR<0.05), several of which are potential short-chain fatty acid producers with anti-inflammatory properties, including taxa from the genus *Subgoligranulum*, and the family Lachnospiraceae. We observed only 1 taxon, belonging to the genus *Bacteroides*, to be enriched among patients in the *low*-dose group. After adjustment for BMI, the results were even stronger with 335 taxa differing by TAC dosing group. Findings were generally similar to unadjusted analyses in that several potentially anti-inflammatory taxa, including the genus *Akkermansia* and family Rumninococcaceae, were elevated in the high TAC dosing group. Relative abundances of the taxa that significantly differed by TAC group in both analyses are displayed in Fig 5. DESEq2 results for the 38 taxa that differed in the unadjusted analysis are displayed in S2 Table.

### Biomarkers of endotoxemia, inflammation and oxidative stress and TAC dosing

Population mean values of LPS, TNF-α and 8,12-iso-isoprostane were 0.43, 1.38 and 94.24, respectively. Above the median daily weight-based TAC dose was associated with higher gut microbial alpha diversity (p = 0.03); concurrently, TNF-α and 8,12-iso-Isoprostane F-2alpha-VI levels were lower and LPS levels were higher in the above median TAC group although these findings were only marginally statistically significant and dependent on BMI adjustment (Table 2). Measures of alpha diversity were inversely correlated with isoprostane and TNF-alpha but positively correlated with LPS, although, despite strong correlations, none of these associations were statistically significant in this small study (Table 3).

**Table 2:** Biomarkers of endotoxemia (LPS), inflammation(TNF-alpha) and oxidative stress (isoprostane) by tacrolimus dosing requirements.

	Low-group ≤ Median TAC Dose		High-group > Median TAC Dose		p-value	
	Crude	BMI adjusted	Crude	BMI adjusted	Crude	BMI adjusted
**Isoprostane**	125.8 (16.6)	118.0 (18.7)	67.2 (15.3)	74.0 (17.0)	0.0251	0.1433
	(n = 6)	(n = 6)	(n = 7)	(n = 7)		
**TNF-alpha**	1.70 (0.21)	1.75 (0.20)	1.07 (0.21)	1.02 (0.20)	0.0536	0.0277
	(n = 8)	(n = 8)	(n = 8)	(n = 8)		
**LPS**	0.38 (0.04)	0.35 (0.04)	0.47 (0.04)	0.49 (0.04)	0.1261	0.0471
	(n = 6)	(n = 6)	(n = 7)	(n = 7)		

Values are presented least squared means (IQR). LPS = lipopolysaccharide, TNF- α = tumor necrosis factor-α.

**Table 3:** Correlation between Shannon index and number of observed ESVs (alpha diversity metrics) and biomarkers of inflammation, endotoxemia and oxidative stress.

	Shannon Index	Observed # ESVs
**Isoprostane** (N = 12)	-0.50, p = 0.10	-0.44, p = 0.15
**TNF-alpha** (N = 15)	-0.48, p = 0.07	-0.50, p = 0.06
**LPS** (N = 12)	0.54, p = 0.07	0.58, p = 0.04

Pearson correlated and p-values presented for rarefied alpha diversity metrics. LPS = lipopolysaccharide, TNF- α = tumor necrosis factor-α.

## Discussion

In the present study, we examined the relationship between the metrics of gut microbial diversity, levels of endotoxemia, inflammation and oxidative stress and TAC dosing requirements after HT. Specifically, we found that higher TAC dosing requirements in the early (within the first 3 months) post-HT period were associated with: 1) greater alpha diversity of gut microbiota, 2) higher abundance of specific taxa with potential anti-inflammatory properties, and 3) lower biomarker levels of inflammation and oxidative stress, and higher endotoxemia, although these latter findings were only marginally statistically significant and dependent on BMI adjustment.

These findings build on prior research from our group that explored the variations in gut microbial communities, endotoxemia and established biomarkers of inflammation and oxidative stress in a large population of HF patients (NYHA Class I-IV) and after HT [9]. We found that advanced HF patients (NYHA Class IV) had reduced levels of gut alpha diversity and heightened levels of endotoxemia, inflammation and oxidative stress relative to less symptomatic HF (NYHA Class I-III) patients. Treatment with HT did not appear to restore gut diversity or improve levels of endotoxemia, while inflammation and oxidative stress were reduced [9].

The gut microbiome is the community of microbes inhabiting the human gastrointestinal tract, which contains nearly 2000 bacterial species [14]. In addition to host elimination pathways (i.e. liver metabolism and renal excretion), the gut microbiota also plays important roles in drug metabolism through secretion of drug-metabolizing enzymes or microbiota-host co-metabolism. While the impact of gut microbiota on drug metabolism has been investigated for decades, data are only available for about 40 drugs or natural products [15]. Previous research has implicated the gut microbiome in the co-metabolism of various non-transplant medications, including acetaminophen, digoxin, simvastatin, and omeprazole [15]. However, data examining the potential impact of the gut microbiome on immunosuppressive medications like TAC in the post-transplant setting are sparse.

Lee et al. evaluated the relationship between the gut microbiome and TAC dosing in 19 kidney transplant recipients [8]. In their analysis, patients who required a 50% increase in TAC dose during the first post-transplant month (n = 5) were compared to patients with stable TAC doses. This study found that the Shannon diversity index was similar between the two groups (3.5±0.8 versus 3.5±0.5; p = 0.78), as were measures of beta-diversity. However, these authors identified that abundance of *Faecalibacterium prausnitzii* was higher in the dose-escalation group when compared to the stable dose patients (11.8 versus 0.8%, respectively; p = 0.002). This finding is relevant as this organism is known to produce a significant amount of butyrate, which is a major energy source for intestinal cells, is readily absorbed by intestinal epithelial cells, and is implicated in the maintenance of colonic mucosal health [16, 17]. This species may also possess anti-inflammatory properties, as a decrease in *Faecalibacterium prausnitzii* has been associated with inflammatory bowel disease [18]. Unfortunately, this study did not evaluate serum inflammatory markers, hence we cannot directly assess if a relationship between gut dysbiosis and systemic inflammation existed in this cohort of renal transplant recipients.

Contrary to the analysis by Lee et al., we identified an association between TAC dosing requirements and alpha diversity as measured by both the Shannon index and the number of observed ESVs. Our positive findings may diverge from Lee et al.’s due to our larger sample size, or from inherent differences in the study design or patient population (i.e. kidney versus HT). Regardless, our study is the first to associate lower TAC dosing requirements with gut dysbiosis in the early post-HT period. In addition to enhanced measures of community diversity, we identified 38 specific taxa that were differentially abundant in patients with higher TAC dosing requirements. Among these is the genus *Subdoligranulum*, which is closely related to *Faecalibacterium prausnitzii* [19]. This finding is relevant in light of the aforementioned salutary properties of *Faecalibacterium prausnitzii*, and it suggests a possible connection between higher abundance of *Subdoligranulum* and the lower levels of inflammation and oxidative stress that we found in our *high*-dose patients.

We posit that several mechanisms may explain the link between gut dysbiosis and TAC dosing requirements post-HT. Global disturbances in the richness and evenness of the gut microbiome—which have been demonstrated in transplant patients with diarrhea—may result in significant derangements in intestinal CYP3A4 and P-glycoprotein activity and subsequent reductions in TAC dosing requirements [5]. It is also possible that increased gut diversity may increase TAC dosing requirements, as drug absorption and/or metabolism may be directly linked to a healthy colonic mucosa requiring butyrate from bacterial sources like *Faecalibacterium prausnitzii* and *Subdoligranulum*. Interestingly, a follow up analysis from the study by Lee et al. [8] found that *Faecalibacterium prausnitzii* can produce a unique metabolite of TAC, which suggests gut bacterial metabolism as a previously unrecognized drug elimination route [20]. While all of these postulates are intriguing, further study is required to elucidate the reasons for the relationship between the gut microbiome and TAC dosing requirements.

Our study has several noteworthy limitations. First, this is a cross-sectional analysis, and longitudinal trends in the relationship between gut microbiome dysbiosis and TAC dosing requirements were not assessed. While our core hypothesis is that the gut microbiota influences the metabolism of TAC, it is also possible that this drug may influence gut microbiota composition as has been suggested in animal models [6,7]. Second, although our two groups were well matched with respect to demographics and background medical history, we did not genotype patients for polymorphisms in CYP3A5, which can significantly alter the pharmacokinetics of TAC. Third, no diet or other behavioral variables were collected among the patients enrolled, hence we cannot determine whether dietary habits contributed to the gut microbiome profile observed in our study. Dietary assessments should be included in future studies aiming to provide knowledge on habitual dietary intake over a longer period. Nevertheless, the likelihood that behavioral variables would have a meaningful influence of TAC dosing is low, thus reducing the potential that unmeasured behaviors confounded our results. Also, we did not administer the Bristol stool scale during stool/blood sample collection; therefore, we are unable to comment on presence or absence of diarrhea and its impact on TAC dosing. However, we wish to point out that diarrhea is likely a consequence (and not a cause) of the altered microbiome. Finally, although this is the largest known dataset of post-transplant patients exploring this topic, we recognize that this is still a pilot study with a relatively small sample size.

In conclusion, we have observed an association between gut alpha diversity and TAC dosing requirements early after HT. Future studies are needed to validate this relationship longitudinally in the post-transplant setting. Additionally, data demonstrating that pre-transplant microbial composition is related to post-transplant TAC dosing requirements would be of high clinical utility. If such an association were established, the pre-operative gut microbiome could be potentially integrated with demographic and genetic variables to better guide post-operative TAC dosing in HT recipients.

## Supporting information

S1 Fig Correlation between rarefied and nonrarefied alpha diversity metrics. (TIFF)

S2 Fig Results for rarefied Alpha diversity metrics and TAC dose groups. (TIFF)

S3 Fig Results for non-rarefied Alpha diversity metrics and TAC dose groups. (TIFF)

S1 Table Timing from transplantation to sample collection dates. (DOCX)

S2 Table Unadjusted and BMI-adjusted DESeq2 results for the ESVs that signififcantly differed in the unadjusted analysis. (DOCX)
