# Supplementary figures and images for: Gut microbial diversity, inflammation, and oxidative stress are associated with tacrolimus dosing requirements early after heart transplantation
Source: PLoS One. 2020 May 29;15(5):e0233646. doi: 10.1371/journal.pone.0233646 (PMC7259664; doi:10.1371/journal.pone.0233646)

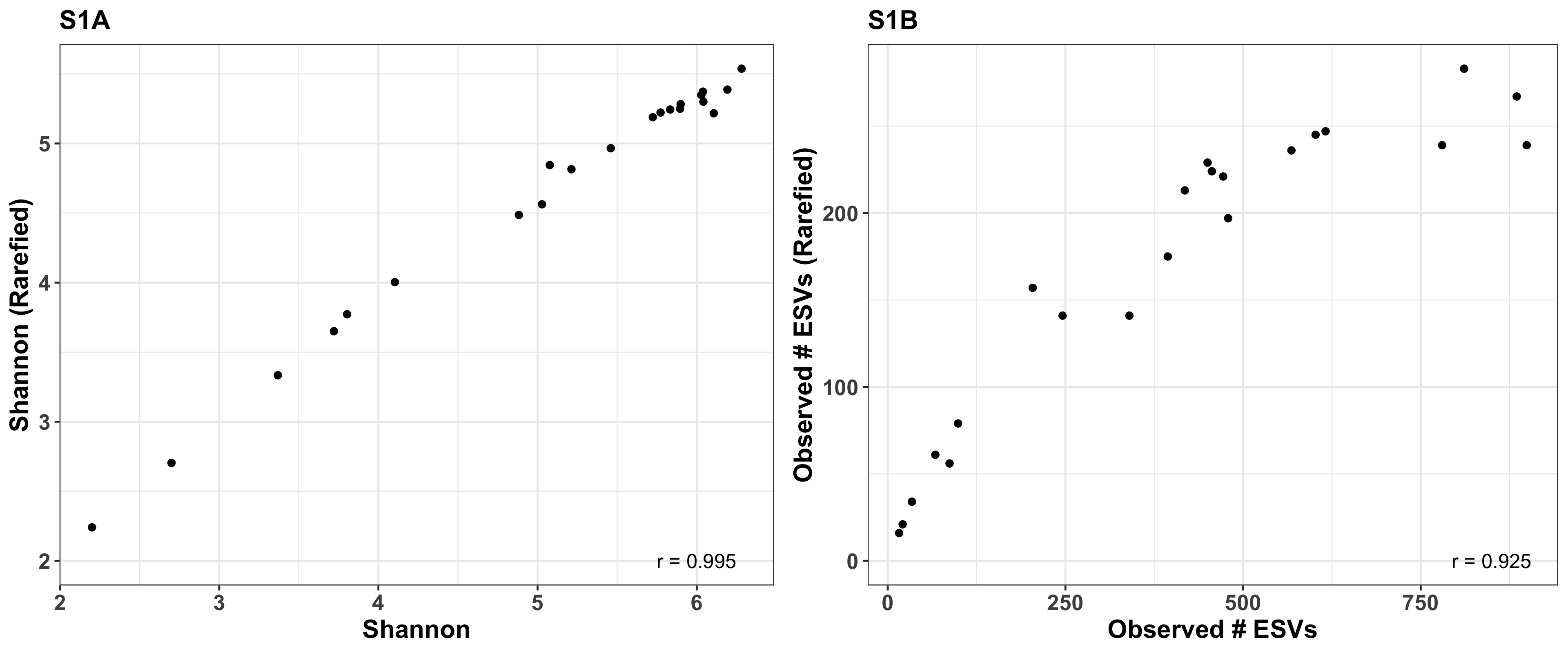

Supplement: S1 Fig — (TIFF) [file pone.0233646.s001.tiff]

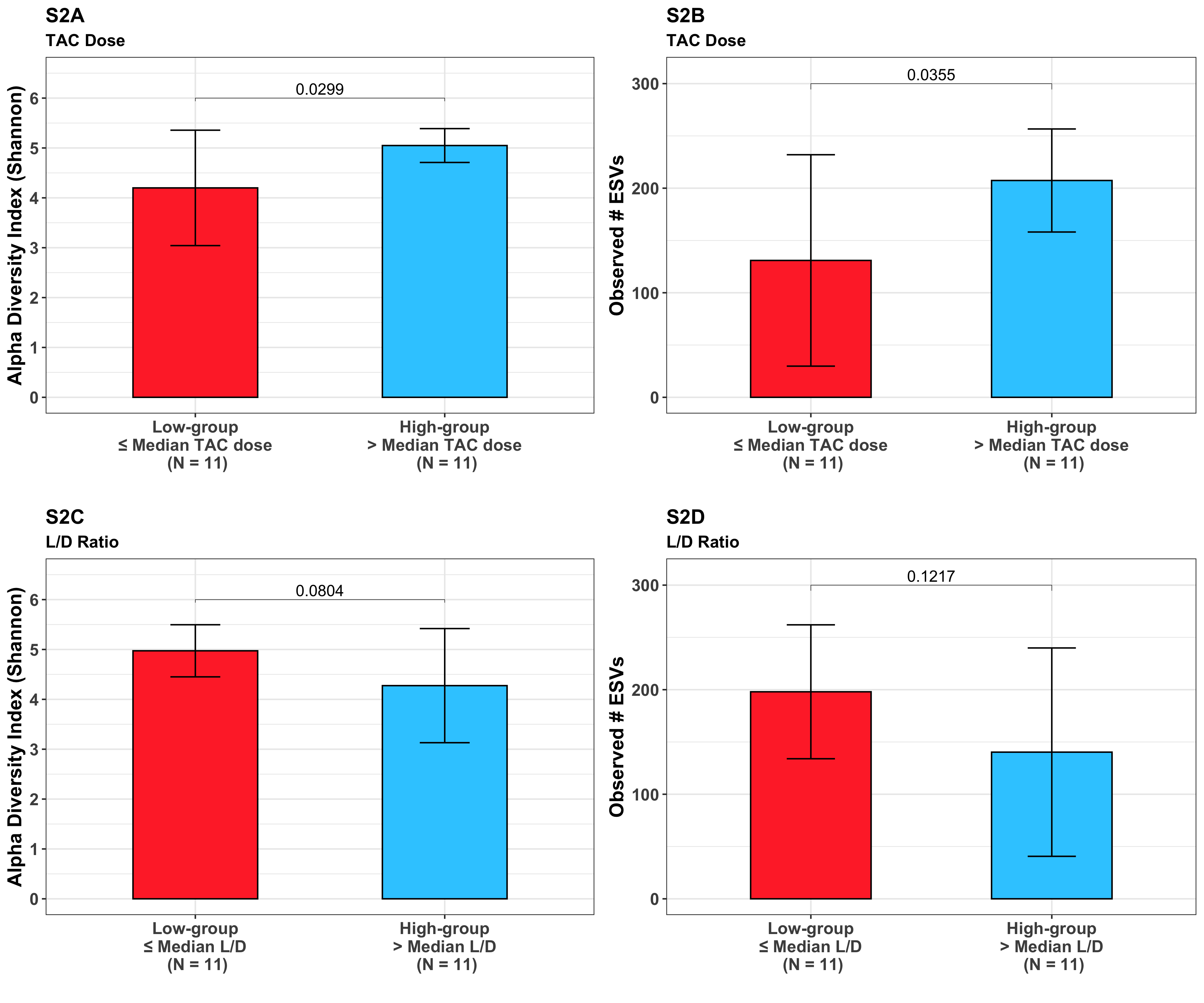

Supplement: S2 Fig — (TIFF) [file pone.0233646.s002.tiff]

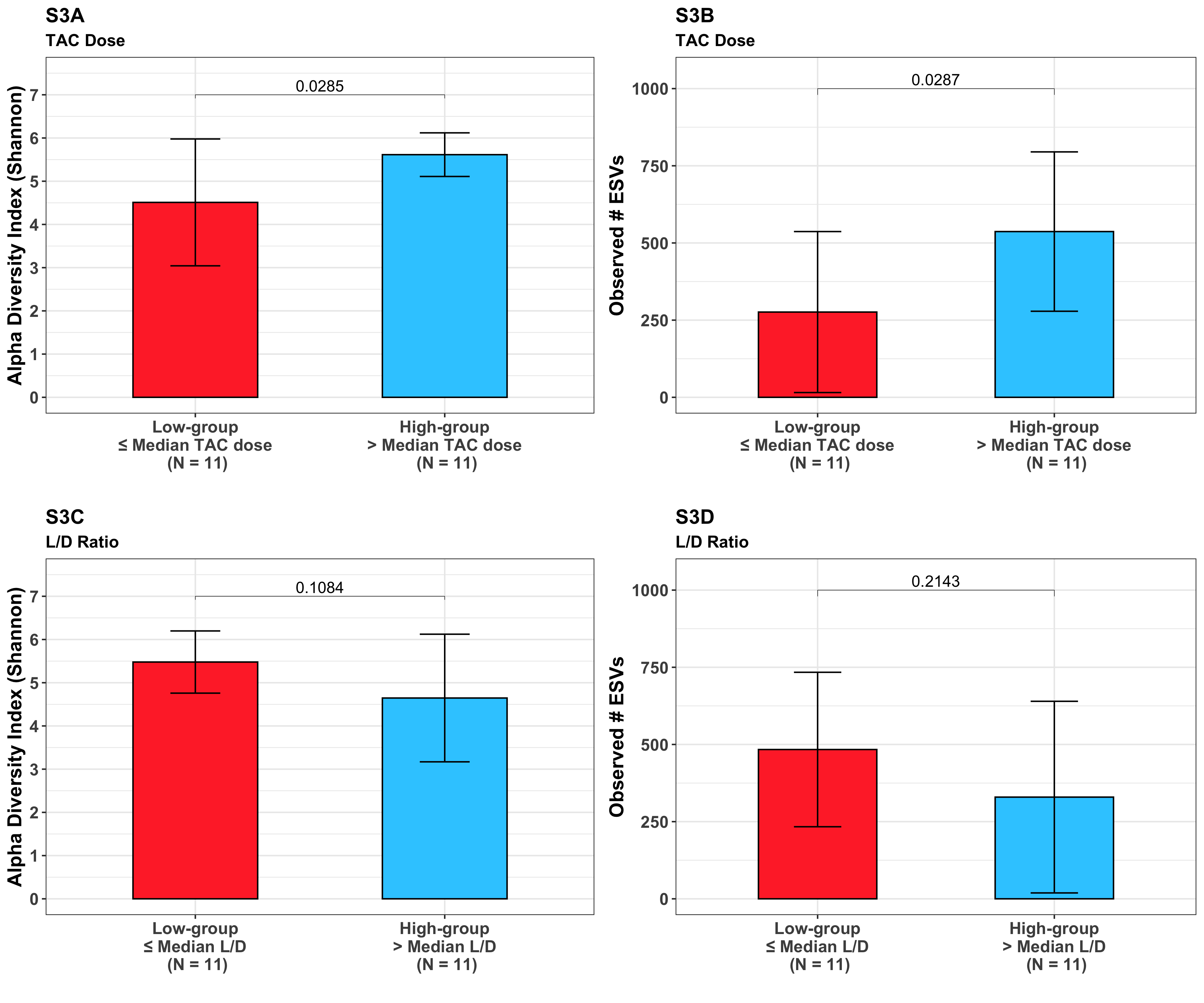

Supplement: S3 Fig — (TIFF) [file pone.0233646.s003.tiff]
