# Supplementary material for: Gut microbial diversity, inflammation, and oxidative stress are associated with tacrolimus dosing requirements early after heart transplantation
Source: PLoS One. 2020 May 29;15(5):e0233646. doi: 10.1371/journal.pone.0233646 (PMC7259664; doi:10.1371/journal.pone.0233646)
Supplement: S1 Table — (DOCX) [file pone.0233646.s004.docx]

**Supplemental Table 1: Timing from Transplantation to Sample Collection Dates**

| **Transplantation to Dose Date** | | | | |
| --- | --- | --- | --- | --- |
|  | **All** | **≤ Median TAC dose** | **> Median TAC dose** | **p-value** |
| **Mean ± SE** | 64.17 ± 6.40 | 66.25 ± 8.55 | 62.08 ± 9.86 | 0.753 |
| **Median** | 54 | 61.5 | 52.5 |  |
| **Min, Max** | 29, 150 | 30, 125 | 29, 150 |  |
| **Transplantation to Level Date** | | | | |
|  | **All** | **≤ Median TAC dose** | **> Median TAC dose** | **p-value** |
| **Mean ± SE** | 61.54 ± 5.93 | 65 ± 8.79 | 58.08 ± 8.23 | 0.572 |
| **Median** | 54 | 61 | 52.5 |  |
| **Min, Max** | 15, 125 | 30, 125 | 15, 116 |  |
| **Transplantation to Stool Sample Date** | | | | |
|  | **All** | **≤ Median TAC dose** | **> Median TAC dose** | **p-value** |
| **Mean ± SE** | 63.46 ± 5.88 | 65.08 ± 8.18 | 61.83 ± 8.78 | 0.789 |
| **Median** | 57 | 61.5 | 55.5 |  |
| **Min, Max** | 30, 131 | 30, 124 | 33, 131 |  |
| **Transplantation to Blood Sample Date** | | | | |
|  | **All** | **≤ Median TAC dose** | **> Median TAC dose** | **p-value** |
| **Mean ± SE** | 56.87 ± 7.50 | 53.62 ± 9.72 | 60.12 ± 11.98 | 0.681 |
| **Median** | 55 | 53.5 | 66 |  |
| **Min, Max** | 17, 111 | 17, 111 | 17, 108 |  |

TAC = Tacrolimus, SE = Standard Error
