# Supplementary material for: Gut microbial diversity, inflammation, and oxidative stress are associated with tacrolimus dosing requirements early after heart transplantation
Source: PLoS One. 2020 May 29;15(5):e0233646. doi: 10.1371/journal.pone.0233646 (PMC7259664; doi:10.1371/journal.pone.0233646)
Supplement: S2 Table — (DOCX) [file pone.0233646.s005.docx]

**Supplemental Table 2: Unadjusted and BMI-adjusted DESeq2 results for the ESVs that signififcantly differed in the unadjusted analysis.**

| **Phylum** | **Family** | **Genus** | **Unadjusted Analysis** | | **Adjusted Analysis^3^** | |
| --- | --- | --- | --- | --- | --- | --- |
|  |  |  | **log2FC** | **p-value^1,2^** | **log2FC** | **p-value^1,2^** |
| **Firmicutes** | **Lachnospiraceae** | **Blautia** | **22.77** | **6.39E-13** | **23.37** | **9.84E-11** |
| **Firmicutes** | **Lachnospiraceae** | **Roseburia** | **20.76** | **3.88E-11** | **23.36** | **9.84E-11** |
| **Firmicutes** | **Lachnospiraceae** | **Anaerostipes** | **22.51** | **9.94E-13** | **23.17** | **9.84E-11** |
| **Firmicutes** | **Lachnospiraceae** | **Tyzzerella_4** | **22.47** | **9.94E-13** | **21.97** | **2.34E-10** |
| **Firmicutes** | **Ruminococcaceae** | **Subdoligranulum** | **22.33** | **1.32E-12** | **19.26** | **7.87E-09** |
| **Firmicutes** | **Ruminococcaceae** | **Subdoligranulum** | **22.04** | **2.26E-12** | **19.14** | **9.88E-09** |
| **Firmicutes** | **Ruminococcaceae** | **Subdoligranulum** | **21.95** | **2.59E-12** | **18.95** | **1.41E-08** |
| **Firmicutes** | **Ruminococcaceae** | **Subdoligranulum** | **8.51** | **1.34E-02** | **8.66** | **1.42E-02** |
| **Firmicutes** | **Lachnospiraceae** | **Uncharacterized** | **8.20** | **9.99E-03** | **7.69** | **1.46E-02** |
| **Firmicutes** | **Lachnospiraceae** | **Uncharacterized** | **8.01** | **1.34E-02** | **7.55** | **1.90E-02** |
| **Firmicutes** | **Lachnospiraceae** | **Uncharacterized** | **7.76** | **1.79E-02** | **7.49** | **2.12E-02** |
| **Firmicutes** | **Lachnospiraceae** | **Uncharacterized** | **8.25** | **1.02E-02** | **7.39** | **2.15E-02** |
| **Firmicutes** | **Lachnospiraceae** | **Uncharacterized** | **7.65** | **2.05E-02** | **7.37** | **2.42E-02** |
| **Firmicutes** | **Lachnospiraceae** | **Uncharacterized** | **7.83** | **1.74E-02** | **7.32** | **2.53E-02** |
| **Firmicutes** | **Peptostreptococcaceae** | **Romboutsia** | **7.09** | **3.69E-02** | **7.20** | **2.83E-02** |
| **Firmicutes** | **Lachnospiraceae** | **Uncharacterized** | **7.44** | **2.73E-02** | **7.05** | **3.51E-02** |
| **Firmicutes** | **Ruminococcaceae** | **Subdoligranulum** | **23.68** | **4.89E-13** | **8.40** | **3.64E-02** |
| **Firmicutes** | **Ruminococcaceae** | **Subdoligranulum** | **23.31** | **4.89E-13** | **8.23** | **4.23E-02** |
| **Firmicutes** | **Ruminococcaceae** | **Subdoligranulum** | **21.56** | **5.54E-12** | **8.16** | **4.47E-02** |
| **Firmicutes** | **Lachnospiraceae** | **Uncharacterized** | **7.19** | **3.69E-02** | **6.79** | **4.68E-02** |
| **Firmicutes** | **Ruminococcaceae** | **Subdoligranulum** | **23.43** | **4.89E-13** | **8.09** | **4.75E-02** |
| **Firmicutes** | **Ruminococcaceae** | **Subdoligranulum** | **21.57** | **5.51E-12** | **8.08** | **4.78E-02** |
| **Firmicutes** | **Ruminococcaceae** | **Subdoligranulum** | **22.20** | **1.65E-12** | **8.07** | **4.82E-02** |
| Firmicutes | Lachnospiraceae | Uncharacterized | 7.22 | 3.68E-02 | 6.64 | 5.28E-02 |
| Firmicutes | Ruminococcaceae | Subdoligranulum | 22.94 | 6.39E-13 | 7.86 | 5.72E-02 |
| Firmicutes | Ruminococcaceae | Subdoligranulum | 23.05 | 6.39E-13 | 7.84 | 5.83E-02 |
| Firmicutes | Ruminococcaceae | Subdoligranulum | 22.76 | 6.39E-13 | 7.61 | 7.03E-02 |
| Firmicutes | Ruminococcaceae | Subdoligranulum | 22.83 | 6.39E-13 | 7.57 | 7.28E-02 |
| Firmicutes | Ruminococcaceae | Subdoligranulum | 22.89 | 6.39E-13 | 7.48 | 7.80E-02 |
| Firmicutes | Ruminococcaceae | Subdoligranulum | 22.78 | 6.39E-13 | 7.41 | 8.22E-02 |
| Firmicutes | Lachnospiraceae | Roseburia | 22.47 | 9.94E-13 | 7.22 | 9.49E-02 |
| Firmicutes | Lachnospiraceae | Blautia | 22.24 | 1.58E-12 | 7.16 | 9.88E-02 |
| Firmicutes | Lachnospiraceae | Blautia | 22.02 | 2.26E-12 | 6.93 | 1.15E-01 |
| Firmicutes | Lachnospiraceae | Blautia | 21.85 | 3.15E-12 | 6.72 | 1.33E-01 |
| Firmicutes | Peptostreptococcaceae | Intestinibacter | 21.80 | 3.50E-12 | 6.63 | 1.42E-01 |
| Bacteroidetes | Bacteroidaceae | Bacteroides | -22.61 | 8.86E-13 | -6.55 | 1.48E-01 |
| Firmicutes | Ruminococcaceae | Subdoligranulum | 21.74 | 3.85E-12 | 3.55 | 5.70E-01 |
| Actinobacteria | Coriobacteriaceae | Collinsella | 22.12 | 1.95E-12 | 0.73 | 9.07E-01 |
| 1. Adjusted for multiple comparisons through FDR. 2. Taxa that significantly differed in both unadjusted and adjusted analysis are in bold. 3. Adjusted for BMI (overweight vs not). | | | | | | |
